# Expression of miR-210 in relation to other measures of hypoxia and prediction of benefit from hypoxia modification in patients with bladder cancer

**DOI:** 10.1038/bjc.2016.218

**Published:** 2016-07-21

**Authors:** J J Irlam-Jones, A Eustace, H Denley, A Choudhury, A L Harris, P J Hoskin, C M L West

**Affiliations:** 1 Translational Radiobiology Group, Institute of Cancer Sciences, University of Manchester, Manchester Academic Health Centre, Manchester M20 4BX, UK; 2 Department of Histopathology, Central Manchester University Hospitals NHS Foundation Trust, Manchester Royal Infirmary, Oxford Road, Manchester M13 9WL, UK; 3 Christie Hospital, Wilmslow Road, Manchester M20 4BX, UK; 4 Molecular Oncology Laboratories, Department of Oncology, Weatherall Institute of Molecular Medicine, John Radcliffe Hospital, Oxford University, Oxford OX3 9DS, UK; 5 Cancer Centre, Mount Vernon Hospital, Rickmansworth Road, Northwood, Middlesex HA6 2RN, UK

**Keywords:** bladder cancer, microRNA, hypoxic modification, necrosis, biomarker, radiotherapy, carbogen and nicotinamide

## Abstract

**Background:**

The addition of hypoxia modifiers carbogen and nicotinamide (CON) to radiotherapy (RT) improved overall survival (OS) in bladder cancer patients in the BCON phase III clinical trial. We investigate whether expression of hsa-miR-210 in BCON patient samples reflects hypoxia and predicts benefit from hypoxia modification.

**Methods:**

In all, 183 T1–T4a bladder cancer samples were available for miR-210 analysis. A total of 86 received RT+CON and 97 received RT alone. TaqMan qPCR plates were used to assess miR-210 expression. Patients were classified as low (<median expression) or high (⩾median) miR-210. Data on other hypoxia biomarkers were available for comparison.

**Results:**

Patients with high miR-210 had a trend towards improved 5-year OS with RT+CON (53.2%) compared with RT alone (37.8% hazard ratio (HR) 1.68, 95% CI 0.95–2.95, *P*=0.07). No benefit was seen with low miR-210 (HR 1.02, 95% CI 0.58–1.79, *P*=0.97). High miR-210 was significantly associated with high HIF-1*α* protein (*P*=0.001), CA9 protein (*P*=0.0004), Glut-1 protein (*P*=0.001), 26-gene hypoxia score (*P*=0.007), tumour necrosis (*P*=0.02) and concurrent pTis (*P*=0.03).

**Conclusions:**

High miR-210 may reflect hypoxia in bladder cancer. However, its ability to predict benefit from hypoxia modification does not improve upon other hypoxia markers. Investigation as part of a miRNA hypoxia signature may reveal the full potential of miR-210.

Muscle invasive bladder cancer has a 5-year overall survival (OS) rate of around 50% despite aggressive management (Kim *et al*, 2000). Conventional treatment involves radical cystectomy or bladder sparing radiotherapy (RT). The BCON phase III clinical trial showed that addition of hypoxia modifiers carbogen and nicotinamide (CON) to RT improved OS (Hoskin *et al*, 2010). Other trials have also shown that patients benefit from fluorouracil (5-FU) and mitomycin C (James *et al*, 2012) or gemcitabine (Choudhury *et al*, 2011). Given the variety of concurrent treatments to add to RT, there is a need for a biomarker to identify patients most likely to benefit from CON. Tumour hypoxia is associated with a poor prognosis in bladder cancer (Hoskin *et al*, 2003; Palit *et al*, 2005; Ord *et al*, 2007; Eustace *et al*, 2013a; Hunter *et al*, 2014). Studies suggest that those with the most hypoxic tumours are most likely to benefit (Eustace *et al*, 2013a; Hunter *et al*, 2014).

MicroRNAs (miRs) are small non-coding sequences of RNA that regulate the production of cellular proteins by either inhibiting mRNA translation, or promoting its degradation. Unlike other RNA types, miRs are not vulnerable to deterioration in formalin-fixed paraffin-embedded (FFPE) samples (Hall *et al*, 2012), and their expression is robust and tightly controlled. There have been many studies showing hypoxia modulation of miRs (Camps *et al*, 2008; Huang *et al*, 2009; Gee *et al*, 2010; Babar *et al*, 2011; Bruning *et al*, 2011). As miR-210 induction is a consistent feature of the hypoxia response (Corn, 2008; Huang and Zuo, 2014), it was chosen for further research as a prognostic and predictive biomarker in this study.

Blick *et al.* recently identified a signature of seven miRs (including miR-210) associated with low oxygen tension in bladder cancer cell lines (Blick *et al*, 2015). To our knowledge there is no study of the hypoxia modulation of miR-210 in bladder cancer tissue. However, there are several studies in other cancer types. Camps *et al.* reported a striking correlation of miR-210 levels with mortality from breast cancer (Camps *et al*, 2008). Gee *et al.* showed that high expression of miR-210 was associated with a poor prognosis in head and neck squamous cell carcinoma (Gee *et al*, 2010). High miR-210 expression can also have a positive prognostic impact in tumour types such as non-small cell lung cancer (Eilertsen *et al*, 2014) and clear cell renal cancer (McCormick *et al*, 2013). Nevertheless, a systematic review and meta-analysis of 16 studies of 7 cancer types, including bladder cancer found that overexpression of miR-210 was associated with poor patient survival (Wang *et al*, 2014).

The aim of this study was to investigate whether miR-210 expression reflects hypoxia and can be used to predict benefit from hypoxia modification in bladder cancer. It was hypothesised that as miR-210 has an important role in the transactivation of HIF-1 target genes involved in tumorigenesis, it may identify hypoxic cancer most likely to benefit from hypoxia-modifying treatment. A retrospective study was carried out to explore whether miR-210 expression predicts benefit from hypoxia modification and to compare miR-210 expression levels with data for other hypoxia markers available from other studies that used the same samples. The samples were taken from patients enrolled in the BCON phase III trial (Hoskin *et al*, 2010).

## Materials and methods

### Patients and tissue samples

REMARK guidelines (McShane *et al*, 2005) were followed throughout. A retrospective study was carried out using FFPE pre-treatment samples obtained from the BCON phase III clinical trial. Patients were diagnosed with histologically proven urothelial (transitional cell) carcinoma of the bladder stage T1–T4a (metastasis free) and randomised between November 2000 and April 2006. Samples were obtained for 251 of the 333 patients enrolled in BCON. A power calculation was performed using survival rates for bladder cancers and published effect sizes for differences between oxygenated and hypoxic tumours from the ARCON trial (Kaanders *et al*, 2002). The calculation assumed a 2-year OS rate of 70% (Hoskin *et al*, 2010) and CON improving survival only in patients with hypoxic tumours. Patients with oxygenated tumours having RT alone or with CON and those with hypoxic tumours having CON would have 70% survival, whilst those with hypoxic tumours having RT alone would have a 40% survival. Analysis of 150 patients would detect this difference in survival with 80% power at a significance level of 0.01. Without bias, 183 samples were selected evenly between the two trial arms in order to fulfil the power calculation allowing for some extra. The study was approved by the Greater Manchester Research Ethics Committee (LREC 09/H1013/24).

Patients received 55 Gy in 20 fractions in 4 weeks or 64 Gy in 32 fractions in 6.5 weeks daily, five times per week. In those randomised to the CON arm carbogen (2% CO_2_ and 98% O_2_) was administered 5 min before and during RT, and an oral dose of nicotinamide (40–60 mg kg^−1^; Larkhall Laboratories, Charlbury, UK) was given to 1½–2 h before each fraction.

### Histopathology

Tissue samples were obtained by pre-treatment transurethral resection of the bladder tumour (biopsy, partial or complete). Tumour debulking was performed using a diathermy loop, which produced strips of tissue approximately 6 mm in width and of viable length. One block per cm tumour diameter was FFPE. One 4 *μ*m haematoxylin and eosin-stained section from each FFPE block was analysed. Staging was both clinical and pathological (TNM AJCC/UICC classifications). Grading was according to the UK Royal College of Pathologists guidelines (WHO, 1973). miR-210 analysis was performed on samples with ⩾10% viable tumour in order to include quantification of stromal miR-210.

### RNA extraction and cDNA synthesis

RNA was extracted from FFPE samples (three 20 *μ*m sections) using the RecoverAll Total Nucleic Acid Isolation Kit (Life Technologies, Paisley, UK), which included DNAse I treatment. RNA purity was assessed using the 260/280 ratio and all samples fell with the range of 1.8–2.1 as recommended by the manufacturer. Additional information on RNA quality assessment is given in the Supplementary Table S1. Total RNA from each sample was reverse transcribed using TaqMan MicroRNA reverse transcription kit (Life Technologies). Preamplification of DNA involved pooled TaqMan assays (miR-210 and miR-16), Preamplification Master Mix (both Life Technologies) and a PCR thermal cycler (Veriti 9902, Life Technologies). PreAmplification Master Mix was validated by the manufacturer to enrich uniformly up to 100 gene-specific targets using 1–250 ng starting material without introducing bias.

### Quantitative PCR for expression of miRNA

Customised 96-well plates were pre-spotted using TaqMan assays by Life Technologies. Quantitative real-time PCR (qPCR) was conducted using the ABI Prism 7900 qPCR system as per the manufacturer's instructions. Manual Cq values were determined using the ABI Prism Sequence Detection System software (Life Technologies). Relative quantification of miR-210 expression was calculated using the 2^−ΔCq^ method (Livak and Schmittgen, 2001). Expression of miR-210 was normalised to the reference gene RPL16. *A priori* it was decided to dicotomise patients as low miR-210 (<median) or high miR-210⩾median) as a median cutoff had been used in other studies using mRNA hypoxia markers (Buffa *et al*, 2010; Eustace *et al*, 2013b) and miR-210 (Camps *et al*, 2008; Gee *et al*, 2010; Osugi *et al*, 2015). Minus reverse transcriptase controls and no template controls were analysed and had negligible Cq values (>38 cycles). Brain reference control RNA (Life Technologies) was included on each plate. Gene expression was within two cycles for all repeats.

### End points and statistical analyses

Analyses were performed using SPSS (IBM, version 12, Portsmouth, UK) and Prism (Graphpad, version 6, La Jolla, CA, USA). All survival analyses were conducted on an ‘intention to treat' basis. Five-year OS time was taken as time from randomisation to death of any cause; patients still alive were censored to date of last follow-up or at 5 years, depending on what was earlier. Five-year local progression-free survival (LPFS) was taken as time to tumour recurrence in bladder, locoregional failure or death from any cause. Patients with persistent muscle-invasive disease or with no cystoscopy post treatment had their time set to zero. Survival estimates were performed using the Kaplan–Meier method and differences compared using the Mantel–Cox log-rank test. Hazard ratios (HRs) and 95% CI were obtained using Cox's proportional hazard model. Heterogeneity in the treatment effect according to miR-210 expression was addressed within a stratified Cox regression model using appropriate stratum-specific treatment variables. The analysis was performed first just with miR-210 and treatment information and second adjusted for prognostic features. The *χ*^2^-test was used to compare proportions across the levels of categorical factors and Yates' correction was used for 2 × 2 tables; the Mann–Whitney *U*-test was used to compare median values for continuous variables between two groups. All *P*-values were two-sided and agreed statistical significance was 0.05. Data for HIF-1*α* (Hunter *et al*, 2014), and CA9, Glut-1 and necrosis (Eustace *et al*, 2013a) were available from other studies. No corrections were made for multiple testing and should be interpreted accordingly. Analyses were carried out for OS and LPFS but as the results were very similar only the results for OS detailed in the text below (LPFS results are shown in Supplementary Figures S1, S2, S4 and S7).

## Results

Tumour sections from 183 patients had a median RNA yield of 88.0 ng *μ*l^−1^ (range 1.1–502.4 ng *μ*l^−1^) and median 80% tumour (10–100%). The samples were preamplified and miR-210 was quantified for all samples (100% success rate). The median miR-210 for the 183 patients was 0.023 (range 1.5 × 10^−3^−0.54).

In the subset of 183 BCON patients selected for study, 97 received RT and 86 received RT+CON. Most patients (181; 99%) received⩾90% of the prescribed RT. In the experimental arm, 80 (93%) patients received⩾90% of the stipulated carbogen doses and 63 (73%) patients received⩾90% of the stipulated nicotinamide doses. All analyses were conducted on an ‘intention to treat' basis. The 183 patients had a median age at randomisation of 75 (51–88) years; 146 (79.8%) patients were male and 37 (20.2%) patients were female. Stage was T1, T2, T3, T4a and T4b in 18 (9.8%), 123 (67.2%), 35 (19.1%), 6 (2.7%) and 1 (0.6%), respectively. All were N0 and M0. There were no statistically significant differences in clinicopathologic features between treatment arms (Supplementary Table S2). Table 1 shows the clinicopathological details in relation to miR-210 expression.

**Figure 1:**
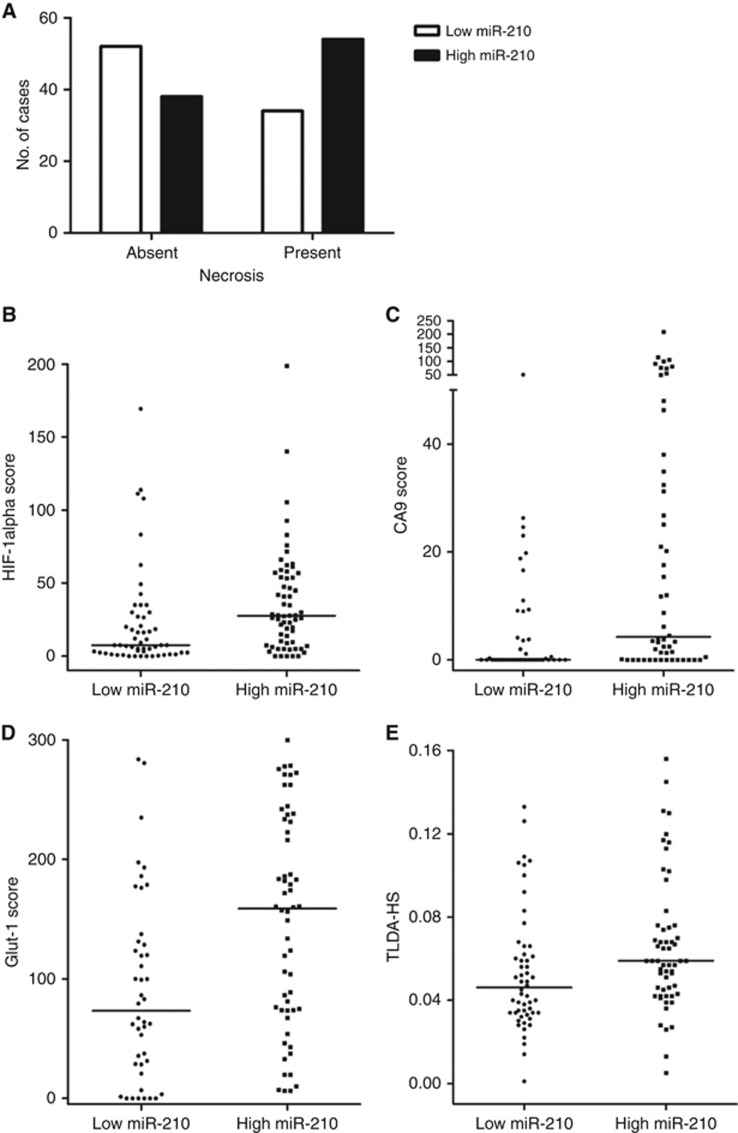
High miR-210 is strongly associated with markers of tumour hypoxia: (**A**) tumour necrosis (*χ*^2^ *P*=0.02); (**B**) HIF-1*α* protein (Mann–Whitney *U P*=0.001); (**C**) CA9 protein (Mann–Whitney *U P*=0.0004); (**D**) Glut-1 protein (Mann–Whitney *U P*=0.001); and (**E**) 26-gene hypoxia score (TLDA-HS) (Mann–Whitney *U P*=0.007). Line represents the median value.

When miR-210 expression levels were compared with data for other hypoxia markers several associations were found. Higher miR-210 expression was observed in patients with high HIF-1*α* (Mann–Whitney *U P*=0.001, *n*=116), CA9 (Mann–Whitney *U P*=0.0004, *n*=98) and Glut-1 (Mann–Whitney *U P*=0.001, *n*=100) protein levels. On the gene level, higher miR-210 expression was associated with a high expression of a 26-gene head and neck cancer hypoxia signature (Mann–Whitney *U P*=0.007, *n*=111). Presence of hypoxia-related tumour necrosis was also associated with high miR-210 expression (*χ*^2^ *P*=0.02, *n*=182). This association was also observed with the presence of concurrent pTis (*χ*^2^*P*=0.03, *n*=183). Expression of miR-210 was significantly associated with per cent tumour material in the sample (*χ*^2^ *P*=0.03, *n*=183). Figure 1 shows the per cent distribution of miR-210 (low *vs* high) according to each hypoxia biomarker. Supplementary Table S3 shows the clinicopathological details per trial arm normalised to % tumour material.

**Figure 2:**
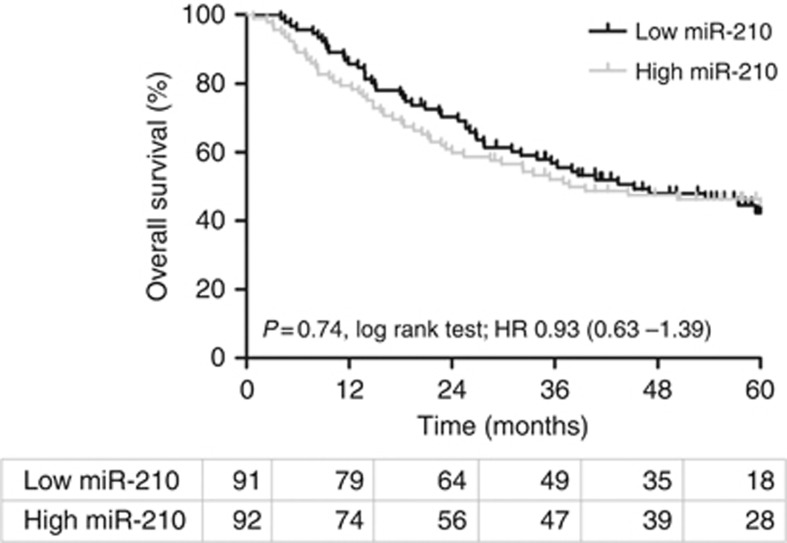
**Kaplan–Meier plot for overall survival according to miR-210 expression in all BCON patients (*n*=183).** Log-rank *P*, hazard ratios (HR) and numbers at risk in each yearly interval are also shown.

Prognosis was investigated in all 183 patients in this study. Figure 2 shows that expression of miR-210 had no prognostic significance for 5-year OS in this patient group (log-rank *P*=0.74). Five-year OS was 46.1% for low miR-210 (<median) and 45.7% for high miR-210⩾median). Of the patient variables only increasing age (*P*=0.004) was associated with a poor prognosis. Table 2 shows the HRs for 5-year OS, and accompanying *P*-values for all clinicopathological variables analysed. Supplementary Table S4 replicates this table using the RT cohort alone.

**Figure 3:**
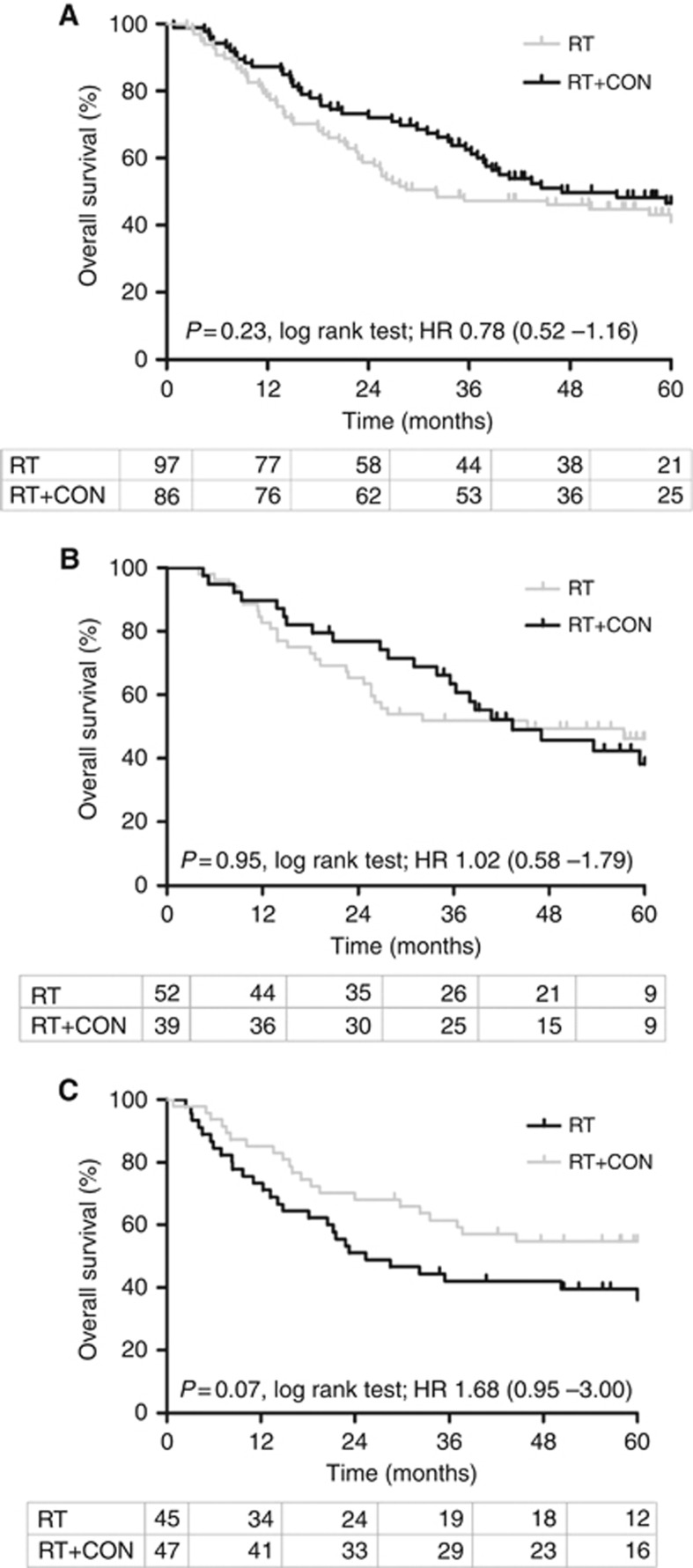
Kaplan–Meier plots for overall survival after radiotherapy (RT) or radiotherapy plus carbogen and nicotinamide (RT+CON) (**A**) without further stratification (*n*=183) and stratified according to (**B**) low miR-210 or (**C**) high miR-210 expression. Log-rank *P*-values, hazard ratios (HR) and number of patients at risk in each yearly interval are also shown.

In the subset of BCON patients studied the benefit of adding CON to RT was not statistically significant (Figure 3A). In the 183 patients studied 5-year OS was 43.3% in the RT arm *vs* 45.3% in the RT+CON arm (log-rank *P*=0.23, HR 0.78, 95% CI 0.52–1.16), which compares with a HR of 0.86 (95% CI 0.74–0.99, log-rank *P*=0.04) in the 333 patients in the BCON trial. When stratified according to median miR-210 expression, there was a trend towards high miR-210 expression predicting benefit from hypoxia modification in patients. In patients with low miR-210 (*n*=91) the 5-year OS was 48.1% for patients receiving RT alone and 43.6% for those receiving RT+CON (log-rank *P*=0.95, HR 1.02, 95% CI 0.58–1.79; Figure 3B). In patients with high miR-210 (*n*=92) the 5-year OS was 37.8% for RT and 53.2% for RT+CON (log-rank *P*=0.07, HR 1.68, 95% CI 0.95–3.00); Figure 3C). The trend towards a reduced risk of death when a patient received RT+CON compared with RT alone in the high miR-210 subgroup was retained in a multivariate analysis including patient age as a covariate (*P*=0.07, HR 1.62, 95% CI 0.92–2.81). This trend was not observed in the low-miR-210 subgroup (*P*=0.96, HR 1.03, 95% CI 0.62–1.84). It is noteworthy that when the data are corrected for % tumour material this trend reaches significance (log-rank *P*=0.04, HR 1.82, 95% CI 1.02–3.23; Supplementary Figures S3 and S4). Survival analyses (OS) were repeated, including patients with samples with ⩾50% viable tumour. Similar trends were seen but with reduced significance due to the smaller number of patients studied (Supplementary Figures S5 and S6).

**Figure 4:**
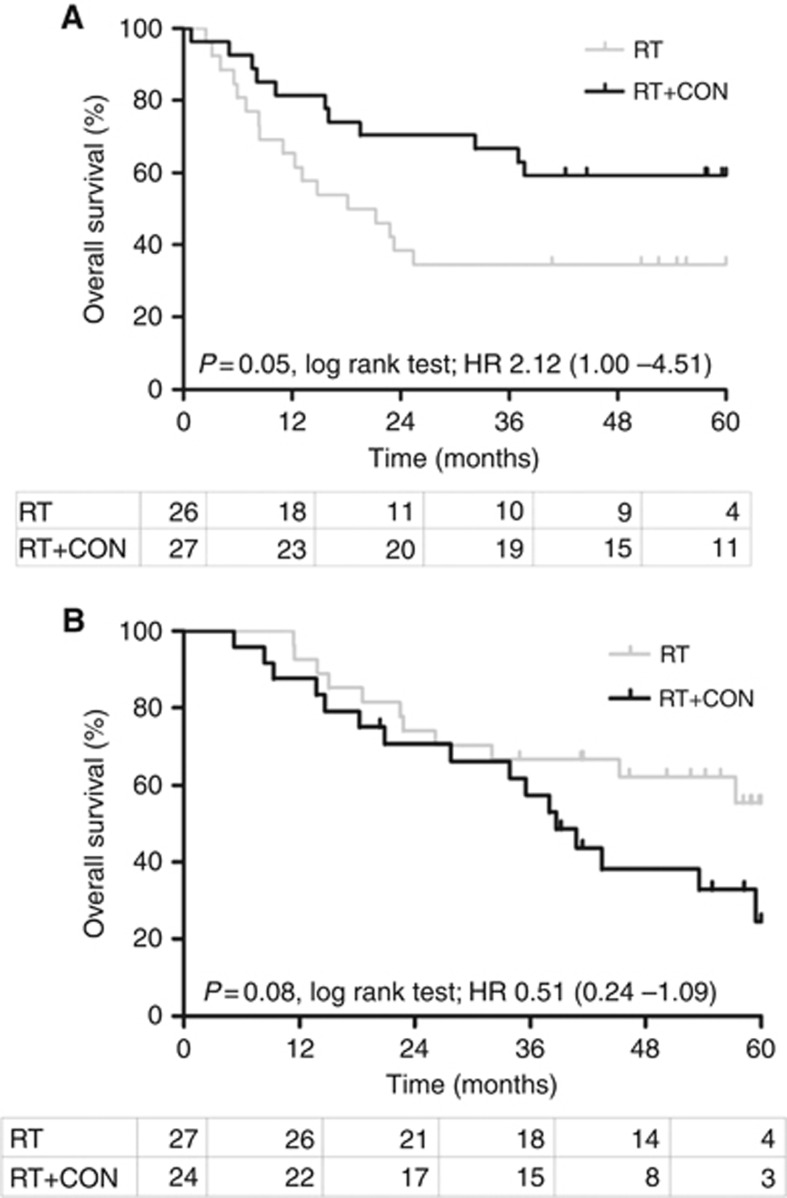
Kaplan–Meier plots for overall survival after radiotherapy (RT) or radiotherapy plus carbogen and nicotinamide (RT+CON) in patients with (**A**) high miR-210 and necrosis present and (**B**) low miR-210 and necrosis absent. Log-rank *P*, hazard ratios (HR) and numbers at risk in each yearly interval are also shown.

Several hypoxia markers can predict benefit from CON. The combination of miR-210 and necrosis, the best independently performing predictor of benefit showed that patients with high miR-210 and necrosis significantly benefit from CON (5-year OS was 34.6% for RT and 59.3% for RT+CON (log-rank *P*=0.05, HR 2.12, 95% CI 1.00–4.51; Figure 4A). The reduced risk of death when a patient received RT+CON compared with RT alone in the high miR-210 and necrosis subgroup retained significance in a multivariate analysis including patient age as a covariate (*P*=0.02, HR 2.31, 95% CI 1.13–4.68). More interestingly, patients with low miR-210 without necrosis show a strong trend that CON treatment may not be suitable (5-year OS was 55.3% for RT and 24.3% for RT+CON, log-rank *P*=0.08, HR 0.51, 95% CI 0.24–1.09; Figure 4B). The age-adjusted HR is 0.53 (*P*=0.10, 95% CI 0.25–1.14).

Five-year LPFS results also show this pattern with significant log-rank results being achieved for both patient populations (high miR-210 plus necrosis *P*=0.02 and low miR-210 without necrosis *P*=0.05; Supplementary Figure S7). The reduced risk of death when a patient received RT+CON compared with RT alone in the high miR-210 and necrosis subgroup retained significance after multivariate analysis (*P*=0.01, HR 2.59, 95% CI 1.26–5.32 *vs P*=0.06, HR 0.50, 95% CI 0.24–1.05 for patients in the low miR-210 without necrosis).

## Conclusions

Tumour hypoxia is associated with poor survival outcomes in bladder cancer (Hoskin *et al*, 2003; Theodoropoulos *et al*, 2004; Palit *et al*, 2005; Ord *et al*, 2007; Eustace *et al*, 2013a; Hunter *et al*, 2014). Tumour hypoxia modification has shown some success in bladder cancer patients treated with RT. The BCON trial showed significant improvements in OS, risk of death and local relapse for bladder cancer patients receiving RT+CON (Hoskin *et al*, 2010). The results of this current study support previous findings that patients with well-oxygenated tumours do not benefit from hypoxia-modifying interventions. In this subset of patients, alternative methods for radiosensitisation are likely to be more effective, and regimens including concurrent 5-FU and mitomycin C (James *et al*, 2012) or gemcitabine (Choudhury *et al*, 2011) should be considered. Other studies have shown that BCON patients with hypoxic tumours benefit most from hypoxia-modifying intervention (Eustace *et al*, 2013a; Hunter *et al*, 2014). We aimed to investigate whether hypoxia-associated miR-210 could improve on the predictive ability of previously studied hypoxia biomarkers. To our knowledge this is the first study of miR-210 as a surrogate marker of hypoxia in bladder cancer tissue.

Direct measurements of tumour hypoxia using an Eppendorf electrode is not feasible in bladder cancer due to tumour inaccessibility. Pimonidazole has been used as a surrogate marker of tumour hypoxia but it too is invasive and its use has not transferred into the clinic. Use of immunohistochemical markers of hypoxia such as HIF-1*α*, CA9 and Glut-1 is very attractive owing to the availability of FFPE material. However, accurate quantification can suffer from analyst subjectivity and sampling bias/tumour heterogeneity. Recent studies have shown the potential of gene signatures to dichotomise head and neck cancer samples according to oxygen status (Eustace *et al*, 2013b) but measurement of mRNA can be limited by its intrinsic instability and presence of RNAses within the cell despite FFPE processing. miRs are more stable than mRNAs and less vulnerable to deterioration in FFPE (Hall *et al*, 2012). We have shown that miR-210 is significantly associated with all hypoxia-related markers available for analysis; HIF-1*α*, CA9, Glut-1, tumour necrosis and expression of a 26-gene head and neck signature.

This study also supports the theory that miR-210 promotes the stabilisation of HIF-1*α* and their expression is interdependent (Chang *et al*, 2013; Wang *et al*, 2014). In hypoxia, miR-210 levels increase in response to binding the HIF-1*α* to the hypoxia responsive element in its promoter region (Corn, 2008), in addition to an increase in nascent primary transcript (pri-miR-210) (Zhang *et al*, 2009). miR-210 also promotes the stabilisation of HIF-1*α* as a part of a positive feedback loop (Chang *et al*, 2013; Wang *et al*, 2014), and its presence is dependent on the level of HIF-1*α*. Future biochemical studies may be able to demonstrate the *in situ* co-localisation of miR-210 and HIF-1*α* in bladder cancer tissue, but this was beyond the scope of this current biomarker study.

miR-210 may be able to stratify patients according to tumour hypoxia and predict benefit from hypoxia modification. There was an improvement in 5-year OS in patients receiving RT+CON compared with RT alone in patients with high miR-210 expression with a significance level of 0.07 following multivariate Cox regression. This difference in treatment success was not observed in patients with low miR-210 expression. It is possible that analysis using a larger cohort of patients may have improved significance levels as our power calculation stipulated 150 patients were required to detect a significant difference in HR with *P*=0.01 and 80% power. However, miR-210 performed less well than other hypoxia markers. Previously, we showed that necrosis, HIF-1*α* and CA9 but not Glut-1 or a 26-gene head and neck signature predicted benefit from the addition of CON to RT in patients with bladder cancer (Eustace *et al*, 2013a, 2013b; Hunter *et al*, 2014). The most predictive markers, and perhaps the best ones for further study, were necrosis, HIF-1*α* and CA9.

When exploring the possibility of combining miR-210 with other hypoxia markers we revealed a significant treatment interaction when miR-210 was combined with necrosis. Patients with a hypoxic tumour were significantly less likely to benefit from CON than patients treated with RT alone. The source of this treatment interaction is unclear, but infers that there is optimal tumour oxygenation status for this treatment type.

In the study reported here miR-210 was not prognostic for OS or LPFS in bladder cancer. A recent systemic review and meta-analysis including 1809 patients suggested that miR-210 had a limited ability as a prognostic factor for OS in several different cancer types including breast and renal cancers (Wang *et al*, 2014). This is consistent with previous findings that HIF-1*α*, CA9 and Glut-1 are not prognostic in BCON patients (Hunter *et al*, 2014), but contradicts results of other studies (Hoskin *et al*, 2003; Palit *et al*, 2005; Ord *et al*, 2007; Eustace *et al*, 2013a). These discrepancies may be due to differences in analytical techniques and sample sizes.

This is the first study to use miR-210 as a biomarker of hypoxia using bladder cancer tissue samples. The results show some potential for miR-210 in this role and support its further examination in an independent cohort of bladder cancer patients using a larger sample size and possibly using a panel of miRs. The median cutoff for miR-210 expression was selected as other authors showed it discriminates patient prognosis (Camps *et al*, 2008; Gee *et al*, 2010; Osugi *et al*, 2015). However, a limitation of this method is that this value is not immediately transferrable between different laboratory sites. In a prospective clinical trial, an initial cohort of patients would need to be analysed to determine the median miR-210 score for subsequent patient classification. Measurement of miR-210 is a simple procedure and most hospital pathology laboratories are equipped to conduct routine qPCR analyses. Use of a clinical test could potentially assist in providing a more personalised treatment plan for patients with bladder cancer. With almost 430 000 new cases diagnosed worldwide in 2012, bladder cancer represents the world's ninth most common cancer (World Cancer Research Fund International, 2014). Use of this biomarker could assist the clinical decision-making process and provide treatment alternatives for patients with hypoxic bladder tumours.

## Figures and Tables

**Table 1:** Clinicopathological details by miR-210 expression

	**Low miR-210 (*****n*****=91)**	**High miR-210 (*****n*****=92)**	***P***
Treatment			
RT	52 (57%)	45 (49%)	0.33
RT+CON	39 (43%)	47 (51%)	
Gender			
Male	76 (84%)	70 (76%)	0.29
Female	15 (16%)	22 (24%)	
Age (years)	75 (53–88)	74 (51–87)	0.94
Tumour stage			
T1	12 (13%)	6 (6%)	0.48
T2	60 (66%)	63 (69%)	
T3	16 (18%)	19 (21%)	
T4	3 (3%)	4 (4%)	
TURBT			
Complete	39 (43%)	39 (43%)	0.92
Partial	27 (30%)	27 (30%)	
Biopsy	23 (25%)	20 (22%)	
No data	2 (2%)	6 (6%)	
% Tumour	75 (10–100)	80 (10–90)	0.03
Necrosis			
Present	38 (42%)	54	0.02
Absent	52 (57%)	38	
No data	1 (1%)	0	
Concurrent pTis			
Present	32 (35%)	18 (20%)	0.03
Absent	59 (65%)	74 (81%)	
Hb (g l^−1^)	13.9 (9.3–17.2)	13.7 (9.8–17.0)	0.20
No data	0 (0%)	1 (1%)	
HIF-1*α* protein	7.5 (0–169.4)	27.7 (0–198.8)	0.001
No data	37 (41%)	30 (33%)	
CA9 protein	0 (0–50.9)	4.3 (0–208.4)	0.0004
No data	47 (52%)	38 (41%)	
Glut-1 protein	73.3 (0–283.8)	158.8 (0–300.0)	0.001
No data	47 (51%)	36 (39%)	
26-gene HS	0.046 (0.001–0.13)	0.059 (0.005–0.16)	0.007
No data	37 (41%)	35 (38%)	

Abbreviations: CA9=carbonic anhydrase 9; CON=carbogen and nicotinamide; Glut-1=glucose transporter-1; Hb=haemoglobin; HS=hypoxia score; pTis=carcinoma *in situ*; TURBT=transurethral resection of the bladder tumour.

Data are represented by *n* (%) or median (range).

**Table 2:** Hazard ratios for 5-year overall survival in all patients

	***N***	**HR**	**95% CI**	***P***
Treatment				
RT	183	1.27	0.86–1.89	0.23
RT+CON				
Gender				
Male	183	0.95	0.58–1.56	0.83
Female				
Median age (years)	183	0.56	0.38–0.83	0.004
Stage				
T1–2	183	1.30	0.81–2.08	0.28
T3–4a				
TURBT				
Complete	176	1.20	0.80–1.80	0.38
Partial/biopsy				
Necrosis				
Absent	182	0.95	0.64–1.42	0.82
Present				
pTis				
Absent	183	1.32	0.84–2.09	0.23
Present				
Median Hb (g l^−1^)	182	1.10	0.74–1.63	0.63
Median miR-210	183	0.93	0.63–1.39	0.74

Abbreviations: CON=carbogen and nicotinamide; Hb=haemoglobin; pTis=carcinoma *in situ*; RT=radiotherapy; TURBT=transurethral resection of the bladder tumour.
