# Supplementary material for: Expression of miR-210 in relation to other measures of hypoxia and prediction of benefit from hypoxia modification in patients with bladder cancer
Source: Br J Cancer. 2016 Jul 21;115(5):571–8. doi: 10.1038/bjc.2016.218 (PMC4997544; doi:10.1038/bjc.2016.218)
Supplement: Supplementary Information [file bjc2016218x1.doc]

**Supplementary Material**

**Table S1. RNA quantification and quality control measurements for BCON FFPE samples. Several typical Agilent Bioanalyzer traces for RIN assessment are show below with the accompanying electrophoresis gel.**

| Samples (N) | RNA yield (ng/µl) | 260/230 ratio | 260/280 ratio | RIN |
| --- | --- | --- | --- | --- |
| 183 | 88.0 (1.1-502.4) | 1.18 (0.32-3.09) | 1.98 (1.89-2.04) | 2.4 (1.0-4.4) |

Data are represented as median (range). Abbreviation: RIN, RNA integrity number.

**Table S2**. Clinicopathological details by trial arm

**Table S3.** Clinicopathological details by miR-210 normalised to % tumour material

**Footnote:** Correction for % tumour was calculated as follows: (miR-210 score/% tumour) x 100.

**Table S4.** Hazard ratios for overall survival in RT patients.

*P*=0.82, log rank test; HR 0.96 (0.65-1.40)

| **Low miR-210 91** | **74** | **62** | **44** | **33** | **13** |
| --- | --- | --- | --- | --- | --- |
| **High miR-210 92** | **65** | **51** | **45** | **37** | **20** |

**Supplementary Figure S1**. Kaplan-Meier plot for local relapse-free survival (LPFS) according to miR-210 expression in all BCON patients (n=183). Log rank *P*, hazard ratio (HR) and numbers at risk in each yearly interval are also shown.

*P*=0.32, log rank test; HR 1.22 (0.83-1.79)

**A**

| **RT 97** | **69** | **54** | **43** | **36** | **15** |
| --- | --- | --- | --- | --- | --- |
| **RT+CON 86** | **70** | **59** | **46** | **34** | **18** |

*P*=0.81, log rank test; HR 0.94 (0.54-1.61)

**B**

| **RT 52** | **41** | **34** | **25** | **20** | **8** |
| --- | --- | --- | --- | --- | --- |
| **RT+CON 39** | **34** | **29** | **21** | **14** | **5** |

*P*=0.14, log rank test; HR 1.52 (0.88-2.65)

**C**

| **RT 45** | **29** | **21** | **19** | **20** | **7** |
| --- | --- | --- | --- | --- | --- |
| **RT+CON 47** | **37** | **31** | **27** | **14** | **13** |

**Supplementary Figure S2.** Kaplan-Meier plots for local progression-free survival (LPFS) after radiotherapy (RT) or radiotherapy plus carbogen and nicotinamide (RT+CON) in (A) all BCON patients (n=183) and stratified according to (B) low miR‑210 or (C) high miR‑210 expression. Log rank *P* values, hazard ratios (HR), and number of patients at risk in each yearly interval are also shown.

**A**

*P* = 0.66, log rank test; HR 0.88 (0.51-1.53), n=91

| **RT 51** | **43** | **34** | **27** | **22** | **10** |
| --- | --- | --- | --- | --- | --- |
| **RT+CON 40** | **36** | **30** | **24** | **14** | **9** |

**B**

*P* = 0.04, log rank test; HR 1.82 (1.02-3.23), n=92

| **RT 46** | **35** | **25** | **18** | **17** | **11** |
| --- | --- | --- | --- | --- | --- |
| **RT+CON 46** | **41** | **33** | **29** | **23** | **16** |

**Supplementary Figure S3.** Kaplan-Meier plots for overall survival after radiotherapy (RT) or radiotherapy plus carbogen and nicotinamide (RT+CON) and stratified according to (A) low miR‑210 (B) or high miR‑210 normalised to % tumour material. Log rank *P* values, hazard ratios (HR), and number of patients at risk in each yearly interval are also shown (n=91).

**A**

*P*=0.56, log rank test; HR 0.85 (0.50-1.46)

| **RT 51** | **40** | **33** | **26** | **21** | **8** |
| --- | --- | --- | --- | --- | --- |
| **RT+CON 40** | **34** | **29** | **20** | **13** | **5** |

**B**

*P*=0.07, log rank test; HR 1.70 (0.96-3.00)

| **RT 46** | **30** | **22** | **18** | **16** | **7** |
| --- | --- | --- | --- | --- | --- |
| **RT+CON 46** | **37** | **32** | **27** | **22** | **13** |

`

**Supplementary Figure S4.** Kaplan-Meier plots for local progression-free survival (LPFS) after radiotherapy (RT) or radiotherapy plus carbogen and nicotinamide (RT+CON) and stratified according to low miR‑210 (A) or high miR‑210 (B) normalised to % tumour material. Log rank *P* values, hazard ratios (HR), and number of patients at risk in each yearly interval are also shown.

**
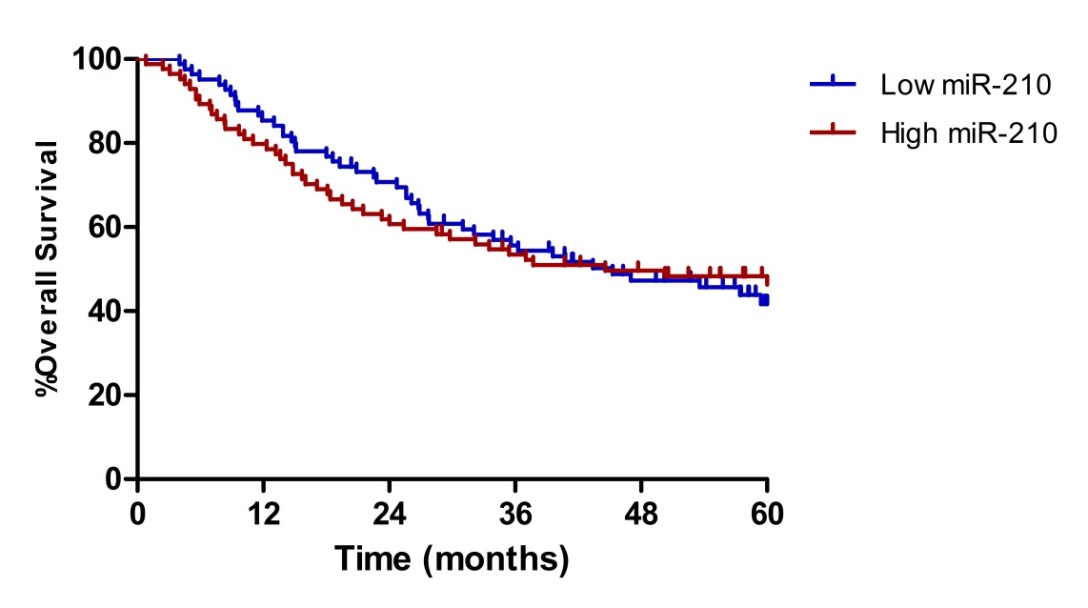
**

*P*=0.98, log rank test; HR 1.00 (0.66-1.51)

| **Low miR-210 82** | **71** | **58** | **43** | **33** | **18** |
| --- | --- | --- | --- | --- | --- |
| **High miR-210 84** | **68** | **52** | **44** | **37** | **26** |

**Supplementary Figure S5**. Kaplan-Meier plot for overall survival according to miR‑210 expression in patient samples with ≥50% viable tumour (n=166). Log rank *P*, hazard ratio (HR) and numbers at risk in each yearly interval are also shown.


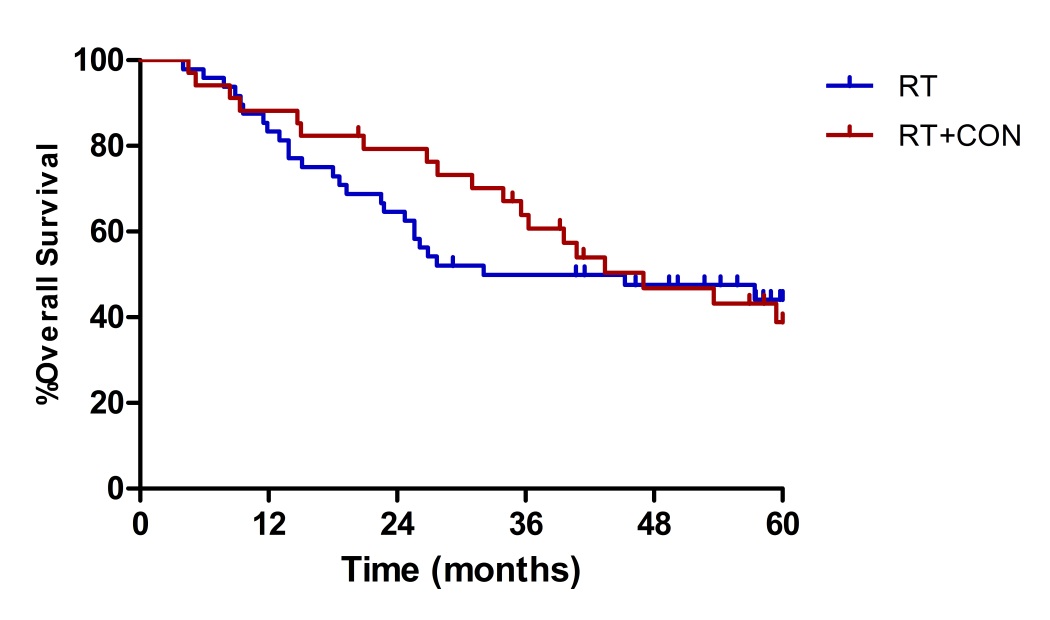


**A**

*P*=0.76, log rank test; HR 1.10 (0.61-2.00)

| **RT 48** | **41** | **32** | **24** | **20** | **9** |
| --- | --- | --- | --- | --- | --- |
| **RT+CON 34** | **31** | **27** | **20** | **14** | **9** |

**B**


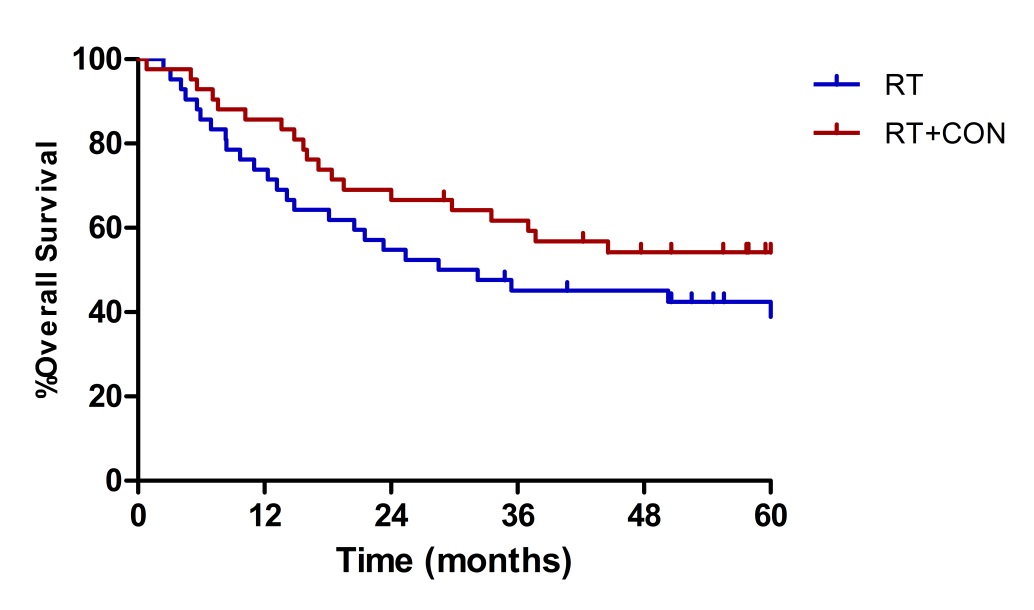


*P*=0.16, log rank test; HR 1.53 (0.84-2.77)

| **RT 42** | **32** | **24** | **19** | **18** | **12** |
| --- | --- | --- | --- | --- | --- |
| **RT+CON 42** | **37** | **29** | **26** | **20** | **14** |

**Supplementary Figure S6.** Kaplan-Meier plots for overall survival after radiotherapy (RT) or radiotherapy plus carbogen and nicotinamide (RT+CON) and stratified according to (A) low miR‑210 (B) or high miR‑210 for patient samples with ≥ 50% viable tumour (n=166). Log rank *P* values, hazard ratios (HR), and number of patients at risk in each yearly interval are also shown.

*P* = 0.02, log rank test; HR 2.51 (1.17-5.39)

**A**

| **RT 26** | **15** | **10** | **10** | **9** | **2** |
| --- | --- | --- | --- | --- | --- |
| **RT+CON 27** | **22** | **19** | **18** | **15** | **8** |

**B**

*P* = 0.05, log rank test; HR 0.46 (0.22-1.00)

| **RT 27** | **23** | **20** | **17** | **13** | **4** |
| --- | --- | --- | --- | --- | --- |
| **RT+CON 24** | **20** | **16** | **11** | **7** | **2** |

**Supplementary Figure S7.** Kaplan-Meier plots for local progression-free survival (LPFS) after radiotherapy (RT) or radiotherapy plus carbogen and nicotinamide (RT+CON) in patients with: A) high miR-210 and necrosis present and B) low miR-210 and necrosis absent. Log rank *P*, hazard ratios (HR) and numbers at risk in each yearly interval are also shown.
